# Objective effect manifestation of pectus excavatum on load-stressed pulmonary function testing: a case report

**DOI:** 10.1186/1752-1947-5-592

**Published:** 2011-12-21

**Authors:** Jason Yongsheng Chan, Wei Liang Huang

**Affiliations:** 1 Ministry of Health Holdings, Singapore; 2 Department of Internal Medicine, Singapore General Hospital, Singapore

## Abstract

**Introduction:**

Pectus excavatum is the most common congenital deformity of the anterior chest wall that, under certain conditions, may pose functional problems due to cardiopulmonary compromise and exercise intolerance.

**Case presentation:**

We present the case of an otherwise physically-adept 21-year-old Chinese sportsman with idiopathic pectus excavatum, whose symptoms manifested only on bearing a loaded body vest and backpack during physical exercise. Corroborative objective evidence was obtained via load-stressed pulmonary function testing, which demonstrated restrictive lung function.

**Conclusion:**

This report highlights the possible detrimental synergism of thoracic load stress and pectus excavatum on cardiopulmonary function. Thoracic load-stressed pulmonary function testing provides objective evidence in support of such a synergistic relationship.

## Introduction

Pectus excavatum, also known as sunken or funnel chest, is the most common congenital deformity of the anterior chest wall, with a prevalence of approximately one in 300 births and a 4:1 male predominance. It is now generally well-accepted that these individuals possess true functional problems due to cardiopulmonary compromise and exercise intolerance, as well as with cosmetic issues alongside possible psychological and social consequences that interfere with quality of life. Furthermore, the condition often deteriorates in late adolescence and early adulthood, with progressive aggravation of the deformity and symptom manifestation [1].

In this paper, we present a case of pectus excavatum in an otherwise physically-adept sportsman, whose symptoms manifested only when he was required to bear a loaded body vest and backpack during physical exercise. The synergistic effect of load-stress and pectus excavatum on cardiopulmonary function is then further discussed.

## Case presentation

Our patient was a 21-year-old Chinese man with a history of congenital idiopathic pectus excavatum without a history of documented cardiac or pulmonary compromise. He was initially seen by the authors reporting of a marked reduction in physical ability whenever he put on a loaded body vest and backpack for training. The attire was a new requirement that arose as a result of a new vocation. At the time of consult, our patient was forthcoming in the description of his symptoms. He had experienced marked dyspnea on exertion, central chest tightness and easy fatigability, which resolved upon removal of the heavy load followed by a few minutes of rest. As a result, he was never able to actively participate in proper training when in full gear, and was never able to keep up with the rest of his colleagues. Prior to this vocation, he was an active sportsman with an outstanding physical condition despite the pectus excavatum defect. His exercise routine included running of more than 30 minutes three or more times per week. His records revealed that he was able to complete a 2.4 km run in 10 minutes whilst in singlet and shorts only. A routine resting pulmonary function test, echocardiography and electrocardiography performed prior to his vocational change did not demonstrate any significant abnormalities (Table 1).

**Table 1:** Physical characteristics of the patient and control subject

	Patient	Control
**Age (years)**	21	21
**Height (m)**	1.74	1.74
**Weight (kg)**	60	60
**Body mass index (kg/m^2^)**	19.8	19.8
**FEV1 predicted**	3.67	3.67
**FVC predicted**	4.33	4.33

**Figure 1:**
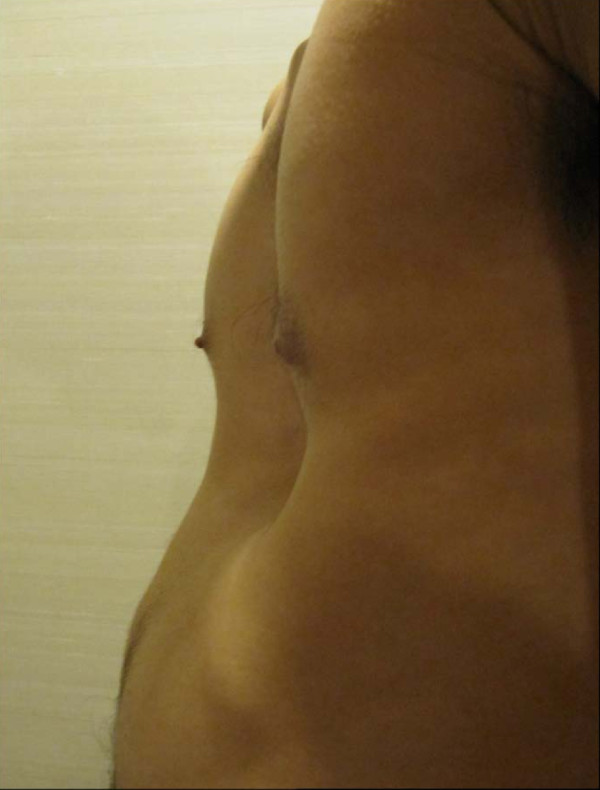
**Chest of our patient with pectus excavatum**.

**Figure 2:**
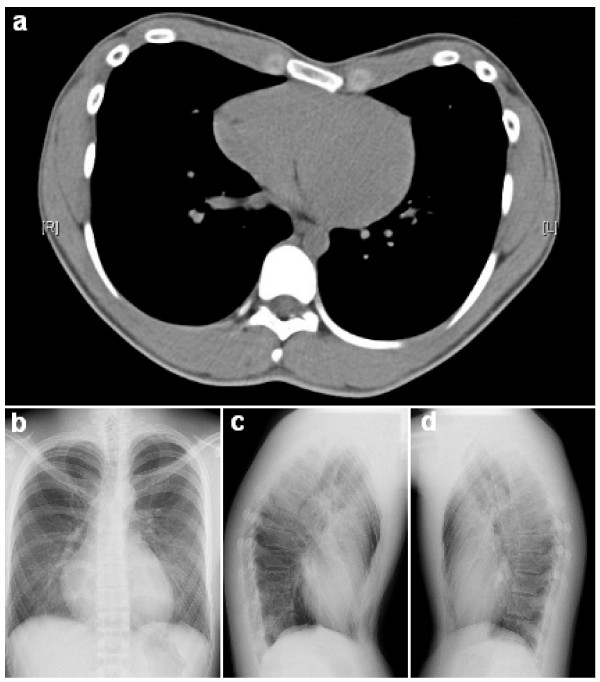
**A Haller index of 2.5 was obtained by dividing the internal transverse distance of the thorax by the sternal-vertebral distance at the most depressed segment of the deformity. (a) **Computed tomography image; **(b-d) **X-ray images.

**Figure 3:**
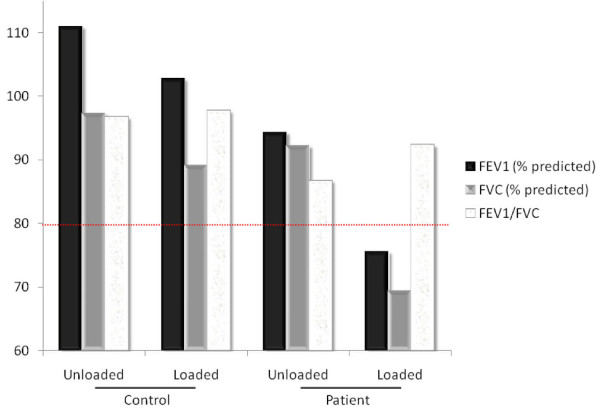
**Static pulmonary indices of our patient and control subject**.

Upon physical examination, the caved-in appearance of his anterior chest was clearly observed (Figure 1). Compensatory anterior flaring of his lower ribs at the costal margins was noted. The apex beat was undisplaced and his heart sounds were normal. No murmurs or other additional sounds were heard. His lung sounds were clear and of equal intensity bilaterally. Plain chest radiographs with anteroposterior and lateral views were subsequently taken, along with computed thoracic tomography (Figure 2). Interestingly, a Haller index of 2.5 was obtained, which is considered to be of mild severity based on such anatomical considerations only. Due to the nature of the precipitating factors (that is, the body vest and backpack) leading to his symptoms, it was highly possible that his chest wall defect compromised his pulmonary function upon load-stress. In order to provide objective evidence to support his symptoms, he agreed to undergo static pulmonary function testing while putting on a body vest and backpack weighing 30% of his body weight. The test was performed on a spirometer (Microlab ML 3500) under close supervision, with the best effort amongst three results recorded. The amount of air which can be forcibly exhaled in the first second (forced expiratory volume in one second, FEV1) and the total amount of air which can be forcibly exhaled after maximal inspiration (forced vital capacity, FVC), were measured. Interestingly, the results showed that, although his pulmonary function at rest was normal when he was in normal work dress (FEV1 94.3%, FVC 92.2%, FEV1/FVC: 86.7), there was a significant deterioration with a restrictive pattern when wearing the body vest and backpack (FEV1 75.5%, FVC 69.3%, FEV1/FVC 92.3). On the other hand, a physically-matched healthy control who voluntarily agreed to undergo the same test did not demonstrate such a drastic deterioration in lung function (Figure 3). Throughout the tests, physical examination and vital signs were otherwise generally unremarkable. A definitive diagnosis of restrictive lung function triggered by load-stress was made in this patient with idiopathic pectus excavatum, and he was subsequently referred to a cardiothoracic surgeon for follow-up.

## Discussion

Pectus excavatum refers to the posterior intrusion of the chest wall into the thoracic cavity. Its occurrence has been documented as far back as the 10^th ^to 16^th ^centuries [2]. Despite its incidence, the etiology of pectus excavatum remains unknown. In a large series in North America, 43% of patients gave a family history of pectus excavatum, demonstrating a genetic component to the disease [3]. Intrinsic abnormality of the costochondral cartilage is suggested by the significant occurrence of pectus excavatum among patients with connective tissue disorders, such as Marfan syndrome, as well as scoliosis [1]. Altered growth of the sternum and costal arches, together with biomechanical factors, are alleged in the pathogenesis (reviewed in [4]). Abnormalities of the cartilages obtained from surgery have also been reported, including stress and/or strain deformation and abnormal collagen content. Such abnormality of the costochondral cartilage may imply a reduced ability to maintain normal work of breathing when the chest wall is restricted by external stressors.

The current standard objective measure of severity of pectus excavatum is the Haller index. This refers to the ratio of the internal transverse diameter of the thorax and the sternal-vertebral distance of the most depressed segment of the deformity. A Haller index of more than 3.25 is associated with moderate-severe disease with cardiorespiratory symptoms. The origin of these symptoms however - whether anatomic, physiologic, or psychological - has long been debated and remains controversial. Two important meta-analyses demonstrated a significant improvement of post-operative cardiopulmonary function along with symptom reduction [5,6], indicating that pectus excavatum is more than just a cosmetic condition.

Whilst most individuals with pectus excavatum are functionally asymptomatic and often demonstrate normal cardiopulmonary tests, the effect of the pectus deformity may manifest only in certain situations or types of exercise performed. Specifically, cardiac output has been shown to be impaired during upright but not supine exercise in patients with pectus excavatum [7]. In addition, other factors which further burden upon cardiopulmonary function can adversely affect these individuals. Internal factors may include the concurrent association of scoliosis, rib hypoplasia or structural heart defects [1], whilst external factors may comprise the type of attire or gear that the individual may be required to bear in certain occupations, such as body vests and backpacks.

Restriction of the chest wall results in reduced expansion during inhalation, thus decreasing the volume of air that the lungs are capable of holding. This may be observed in certain occupational situations which involve the need to put on body vests, body armor [8] or backpacks [9,10], and is often featured in certain forms of physical deformities, including pectus excavatum. Although most patients with uncorrected pectus excavatum are clinically asymptomatic and frequently exhibit normal cardiopulmonary tests, a severely concave chest wall may cause significant lung compression, mediastinal shift and cardiovascular displacement, resulting in cardiopulmonary impairment and poor exercise tolerance [11].

In our case report, although the affected individual with pectus excavatum displayed excellent exercise capability, the underlying respiratory mechanics were most likely altered in order to compensate for the chest wall deformity. The increased work of breathing may in turn imply an inability to increase his ventilatory function further, as normal subjects can, when the chest wall is burdened by an added elastic load [12]. It is known that load carriage induces a limitation on the ventilatory pump that mimics restrictive lung diseases. Pulmonary function testing while standing upright and carrying a backpack weighing 35% to 42% body weight demonstrated a 5% to 7% decrement in both FVC and FEV1 [9,10] in normal healthy individuals. In addition, the magnitude of reduction of pulmonary function was related to the style of load carriage system used. Generally, the greatest decrements were caused by those which covered the entire trunk [9]. Such chest wall restrictive devices provide an added inspiratory load in the breathing cycle that results in a decline in pulmonary function representative of those seen in restrictive pulmonary diseases [8]. In our patient, the load stress of 30% body weight resulted in an approximately 20% reduction in FVC and FEV1, compared to 8% in the healthy subject. The restriction caused by the concurrent carriage of both the backpack and body vest in this case created a circumferential chest wall-restrictive load, which in turn inhibited his inspiratory volume because his chest wall expansion was hampered. The accelerated development of respiratory muscle fatigue, as would be expected with physical activity, would thus limit exercise performance. Such a synergistic effect of load-stress on lung function in pectus excavatum is reminiscent of a previous study which showed that pulmonary function in schoolgirls with adolescent idiopathic scoliosis were more sensitive to backpack load compared with healthy girls [13].

It is noteworthy that load-stressed pulmonary function testing provided corroborative objective evidence in support of our patient's subjective complaints. Such a test may provide a simple, non-invasive, objective tool to evaluate exercise tolerance, especially in an occupational setting where heavy loads or body vests are needed to be borne. In addition, it indicates a true physiologic rather than completely psychological basis for the symptoms. Perhaps more importantly, it may be used in the consideration for corrective surgical procedures, as well as in the evaluation of pre- and postoperative lung function. In a meta-analysis, Malek *et al. *concluded that surgical repair of pectus excavatum does not significantly improve pulmonary function, but ingeniously added that this may be 'a result of testing... under conditions in which pectus excavatum does not manifest itself' [6]. Perhaps a better way to evaluate pectus excavatum will be to assess the altered respiratory mechanics, and consequently reduced pulmonary reserve, which can manifest during static or even exercise thoracic load-stress tests.

## Conclusion

The manifestation of asymptomatic pectus excavatum may be conditional upon the presence of thoracic load stress. Thoracic load-stressed pulmonary function testing provides corroborative objective evidence in support of such a synergistic relationship.

## Consent

Written informed consent was obtained from the patient for publication of this case report. A copy of the written consent is available for review by the Editor-in-Chief of this journal.

## Competing interests

The authors declare that they have no competing interests.

## Authors' contributions

CJY analyzed and interpreted the patient data regarding the condition, and wrote the first draft of the manuscript. HWL performed the lung function tests under the specific conditions mentioned, and was a major contributor in writing the manuscript. Both authors read and approved the final manuscript.
